# An Albumin-Derived Peptide Scaffold Capable of Binding and Catalysis

**DOI:** 10.1371/journal.pone.0056469

**Published:** 2013-02-22

**Authors:** Immacolata Luisi, Silvia Pavan, Giampaolo Fontanive, Alessandro Tossi, Fabio Benedetti, Adriano Savoini, Elisa Maurizio, Riccardo Sgarra, Daniele Sblattero, Federico Berti

**Affiliations:** 1 Dipartimento di Scienze Chimiche e Farmaceutiche, Università di Trieste, Trieste, Italy; 2 Dipartimento di Scienze della Vita, Università di Trieste, Trieste, Italy; 3 T&B Associati srl., Area Science Park, Trieste, Italy; 4 Dipartimento di Scienze della Salute, Università del Piemonte Orientale “Amedeo Avogadro”, Novara, Italy; University of Edinburgh, United Kingdom

## Abstract

We have identified a 101-amino-acid polypeptide derived from the sequence of the IIA binding site of human albumin. The polypeptide contains residues that make contact with IIA ligands in the parent protein, and eight cysteine residues to form disulfide bridges, that stabilize the polypeptide structure. Seventy-four amino acids are located in six α-helical regions, while the remaining thirty-seven amino acids form six connecting coil/loop regions. A soluble GST fusion protein was expressed in *E. coli* in yields as high as 4 mg/l. This protein retains the IIA fragment’s capacity to bind typical ligands such as warfarin and efavirenz and other albumin’s functional properties such as aldolase activity and the ability to direct the stereochemical outcome of a diketone reduction. This newly cloned polypeptide thus represents a valuable starting point for the construction of libraries of binders and catalysts with improved proficiency.

## Introduction

In recent years, a number of different binding proteins have been proposed as alternatives to conventional antibody-based technologies. Affibodies [1], anticalins [2], knottins [3] and neocarzinostatin-based [4] peptide scaffolds are successful examples of protein-protein or protein-peptide recognition systems [5]–[7]. However, the recognition of small molecules by peptides and proteins is more demanding. The engineering of novel protein scaffolds for binding small molecules has several drawbacks common also to antibody-based technologies. Both recombinant anti-hapten antibodies selected from *in vitro* display technologies and artificial protein receptors have, in general, a moderate affinity for small cognate molecules reflected by K_d_ values in the micromolar range, comparable to those of the primary IgM immune response in animals. Obtaining higher-affinity antibodies or artificial receptors against haptens is still a challenge. Multiple “affinity maturation” approaches have been attempted [8]. As a general strategy, large, full-length gene libraries or, alternatively, focused optimized scaffolds can be constructed. In both cases, specific selection protocols must be devised, using modified haptens to optimize presentation to the immune system or to synthetic libraries. Usually, small haptens are chemically modified at a suitable functional group to introduce a reactive linker for conjugation to a carrier protein or immobilization on a solid surface, often via biotinylation. This modification however reduces the number of available interactions and constrains the orientation of the small ligand with respect to potential receptors. Furthermore, the linker and/or the carrier system can elicit a high level of cross reactivity, lowering the affinity for the free, unmodified hapten. A detailed example has been reported involving a testosterone-binding peptide derived from neocarzinostatin [9].

Peptide scaffolds, intermediate between large enzymes and small organocatalysts, have also been proposed as tailor-made catalysts for organic reactions [10]. In most cases, however, successful catalysts are synthetic oligopeptides with notably short sequences of amino acids that only play an ancillary role to the main organocatalytic residue side chain (often acting by covalent catalysis) without forming a site for selective substrate recognition [10]. Tanaka and Barbas used phage display to obtain libraries of more structured peptides exhibiting aldolase activity [11], [12]. However, a general strategy for developing libraries of structured catalytic peptides that can control regio- and stereoselectivity in a wider variety of reactions has not yet been defined.

Being interested in the development of artificial peptides that can efficiently combine binding and catalysis, we reasoned that a convenient starting point would be a “wild-type” sequence that is capable of both recognizing a broad spectrum of small molecules with micromolar affinity and catalyzing a wide range of reactions with acceptable proficiency. Such a peptide could be regarded as an “artificial IgM,” and the possible development of a library of binders by random or rational mutation strategies could mimic the “*in vivo*” antibody affinity maturation process in a single step. As a first contribution towards such ambitious goal, we have carried out a study on a peptide deriving from human serum albumin (HSA). Serum albumin is a promising candidate due to its ability to bind a variety of small molecules with micromolar affinity [13], and to control the outcome of certain chemical reactions. Broad binding activity and chemical reactivity are associated with the binding site located in the albumin IIA subdomain [14], also known as the Sudlow site I. A lysine residue is present in this binding site in both human and bovine serum albumin (K199 and K222 in HSA and BSA, respectively). In addition to being a site for covalent interactions with drugs such as aspirin and benzylpenicillin [15], this basic residue, which is surrounded by a hydrophobic environment, is responsible for the ability of albumin to behave as an enzyme-like catalyst in reactions such as β-eliminations [16], the decomposition of Meisenheimer adducts [17], and the Kemp elimination [18], [19]. Albumin’s ability to direct the stereoselective reduction of diketones and catalyze the aldol reaction has been recently reported by us [20]–[22]. Furthermore, a tryptophan residue (W214) located at the bottom of the main hydrophobic subsite can be exploited as a useful internal fluorescent sensor for binding of different ligands to this site [23], [24]. This could allow the selection of binders in rapid screening protocols that do not require labeling or immobilization of the target ligand. In this report we describe the identification, cloning as a soluble GST fusion protein and characterization of a 101-amino-acid polypeptide derived from the sequence of the IIA binding site of human albumin. This protein retains the ability of native albumin to bind typical ligands, such as warfarin and efavirenz, and to catalyze or control chemical reactions such as the aldol reaction and the reduction of diketones. These results are promising in the view of using this peptide as the base sequence for building libraries of stable artificial receptors and catalysts that can be easily selected for sensing and synthetic applications.

## Results and Discussion

### Identification, Cloning and Production of the GST-HSA100 Polypeptide

**Figure 1:**
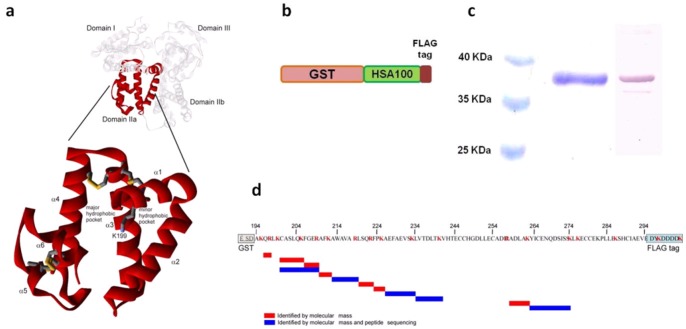
Structure of the human serum albumin HSA-100 fragment. (**a**) The full-length protein and HSA-100 fragment are shown as white and red ribbons, respectively. The structure was obtained from the Protein Data Bank (1BKE). (**b**) Designed construct bearing the polypeptide GST-HSA100. (**c**) SDS-PAGE and anti-FLAG tag Western blot of purified GST-HSA100 protein. (**d**) Tryptic map of GST-HSA100. Tryptic peptides obtained by GST-HSA100 digestion were analyzed by LC-MS/MS. Only peptides covering the GST-HSA100 sequence are shown. Their identities were assigned on the basis of molecular mass (red bars) or peptide sequence (blue bars).

Based on structural and functional analyses, we have identified a peptide corresponding to a 101-residue stretch of the human serum albumin sequence (A194 to E294) named HSA100 (Fig. **1a**). This fragment corresponds to approximately half of albumin’s subdomain IIa, containing Sudlow site I. The binding site is formed by a continuous sequence of amino acids with no contribution from residues from other subdomains. The HSA100 sequence includes all the residues that contact typical albumin ligands, such as warfarin [25], as well as eight cysteine residues that are involved in the formation of the four disulfide bridges (C200–C246, C245–C253, C265–C279, C278–289) of the IIA subdomain. Seventy-four amino acids are located in six α-helical regions (α1, Q196–F206; α2, R209–R222; α3, A229–H247; α4, L250–S270; α5, L284–G292; α6, L284–G292), and the remaining residues are located in six connecting coil/loop regions. A molecular dynamics simulation was performed at room temperature using the Gromacs package and demonstrated that HSA100 is conformationally stable for over 1000 ns simulation.

To simplify production of the HSA100 fragment we decided to clone and express its coding sequence in *E. coli*. A preliminary evaluation of protein production was performed by fusing the HSA fragment to either a carrier maltose-binding protein (MBP) or different tag sequences (HIS6 and strep tags). Even though HSA100 was expressed, purified, and active, the protein yield was extremely low (data not shown). To improve the yield, we used a two step approach: first the wild type HSA100 DNA sequence was modified using an optimization strategy to maximize both transcription and translation efficiency [26]. GC content was optimized, RNA secondary structure and killer motifs were eliminated and codon usage was set for *E. coli* expression. After chemical synthesis the DNA the fragment coding for HSA100 was cloned as a fusion protein with a glutathione S-transferase (GST) carrier (Fig. **1b**), generating the GST-HSA100 fusion. In the second step protein production and purification were optimized by testing a number of different parameters: bacterial strains, temperature of growth, induction condition, and protein extraction protocol. By using the best conditions we managed to produce 15–20 mg/l of GST-HSA100 fusion protein and obtain up to 4 mg/l of soluble protein. The soluble fraction was purified by affinity chromatography using a GSH resin, resulting in highly pure, stable, full-length protein (Fig. **1c**).

HSA100 is the shortest functional peptide that has ever been derived from the HSA sequence and, to the best of our knowledge, the first albumin fragment successfully expressed in *E. coli*. In the past decade, several larger fragments have been described. To define the structural elements required for the formation of the warfarin binding site, Rüker and colleagues prepared five fragments, including domains I, II, I-II, I-IIa and Ib-II [27], [28], [29]. Similar work was completed by the East group on fragments corresponding to domains I-II and II-III [30]. More recently, a recombinant protein corresponding to domain I-II was designed by Fasano [31] to study the allosteric linkage between the warfarin and heme binding sites in the protein. Three recombinant full domains of HSA have also been prepared as potential drug delivery tools [32]. All the described fragments, including native HSA, have been obtained as secreted proteins by expression in *Pichia pastoris*. Using a combined strategy of optimizing DNA sequences and growth conditions, we were able to express and purify soluble, full-length GST-HSA100 protein in *E. coli*.

### Structural Characterization of GST-HSA100

LC-MS/MS and peptide sequencing analyses were performed to confirm the identity of GST-HSA100. Purified GST-HSA100 was trypsin-digested, and the resulting peptides were separated by RP-HPLC. The identities of the peptides were confirmed by comparison of theoretical and experimental *m*/*z* values of full-length peptides (MS) and collision-induced dissociation (CID) fragments (MS/MS). The tryptic peptide map referring to the GST-HSA100 sequence is shown in Figure **1d**, and the mass spectrometry data are summarized in Table S1. Mass spectrometry in combination with SDS-PAGE and anti-FLAG immunoblotting unambiguously demonstrated the correct production of a full-length recombinant soluble GST-HSA100 fusion construct.

**Figure 2:**
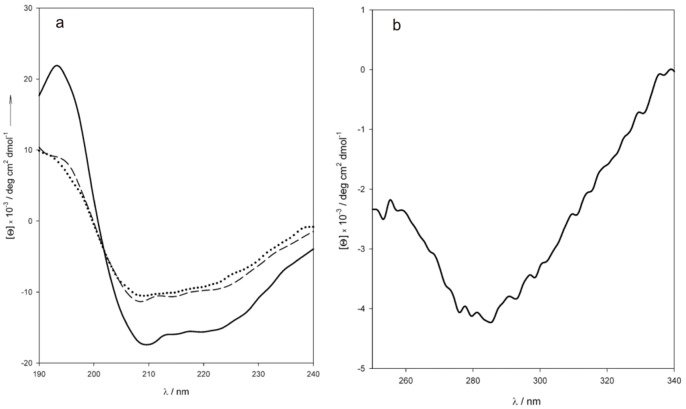
Circular dichroism of GST-HSA100. (**a**) Far-UV CD spectra. Solid line, 25°C; dashed line, 60°C; dotted line, 75°C. The spectra were recorded at 0.2 µM protein in 10 mM phosphate buffer, pH 7.4. (**b**) Near-UV CD spectrum of GST-HSA100.

The circular dichroism spectra of the fusion protein in the near- and far-UV regions (Figure **2** **)** were recorded at neutral pH and temperatures ranging from 25 to 75°C. The far-UV spectrum (Fig. **2a**) was deconvoluted using the convex constraint algorithm [33] to reveal a protein composition of ∼50% α-helix, 30% coil, 16% turn and <5% β-sheet. The secondary structure composition inferred from the CD data is consistent with a sequence that contains the helices and six coil/loop region of the HSA100 domain and two GST domains with a βαβαββα folding topology along the first 84 residues followed by an extended α-helical domain of 132 amino acids at the C-terminus [34]: the expected composition of the fusion protein is 55% helix, 25% coil, 15% turn and 5% sheet. Some thermal denaturation occurred above 60°C, but even at 75°C the conserved amount of α-helix was still over 40%, with no clear trend towards complete unfolding (Fig. **2a**). The thermal stability of the GST-HSA100 protein is comparable to those of HSA and GST [35], [36]. The presence of disulfide bonds was probed by near-UV CD spectroscopy (Fig. **2b**). Several peaks were identified in the fine structure: the shoulder at 267 nm and minima at 276, 284 and 293 nm resemble a set of peaks attributed to the presence of disulfide bridges in the chiral environment of domain II of HSA [27]. The peaks disappear upon reduction by heating the protein in the presence of excess β-mercaptoethanol, and this confirm their attribution to disulfide bridges. None of the four cysteine residues in GST is believed to be involved in disulfide bond formation, even in the predominant homodimer that forms at micromolar concentrations [34], [37], [38], and the observed peaks should be therefore related to disulfide bridges in the HSA region of the protein. However, the mass spectrometry analysis shows the presence of peptides including reduced C200 and C289 indicating that, in at least a fraction of the protein, these two cysteines are not involved in disulfide formation probably because of the exposition of the protein to the very large excess of glutathione during the purification step. On the other side, peptides containing C245, C246, C253 and C265 C278, C279, are missing in the tryptic map: this is an indirect evidence for the presence of disulfide bridges within the main loops of the HSA region, as regions containing disulfides are known to be more rigid thus conferring resistance to trypsin proteolysis [39].

### Small Molecule Binding Activity of GST-HSA100

**Figure 3:**
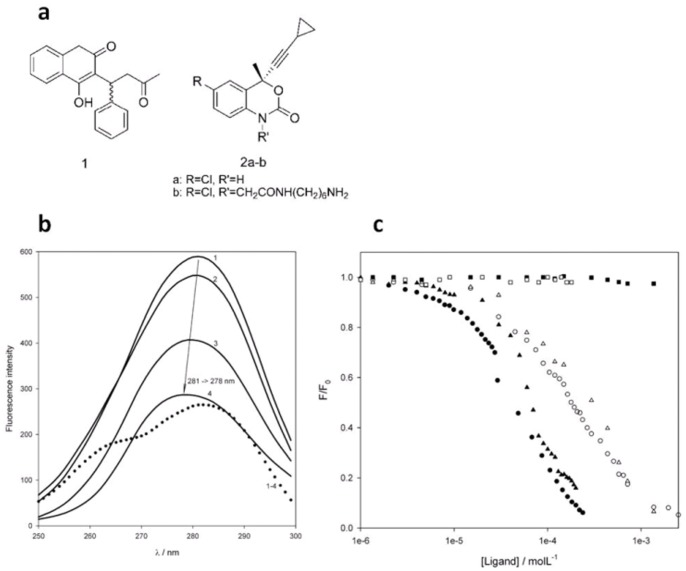
GST-HSA100 small ligand binding. (**a**) Reference albumin IIa site ligands warfarin (1), efavirenz (2a) and efavirenz with amine linker (2b). (**b**) Synchronous (Δ = 60 nm) fluorescence spectra of GST-HSA100 1 µM in 10 mM phosphate buffer, pH 7.4. 1, no quencher added; 2, 10 µM efavirenz; 3, 100 µM efavirenz; 4, 200 µM efavirenz; and 1–4 (dotted line), difference between spectrum 1 and 4. (**c**) Titration of fluorescence emission of HSA, GST-HSA100 and GST by warfarin and efavirenz. •, warfarin-HSA; ▴, warfarin-HSA100; ▪, warfarin-GST; ○, efavirenz-HSA; Δ, efavirenz-HSA100; □, efavirenz-GST.

(±)-Warfarin and efavirenz (Figure **3a**) were chosen as probes to test the binding capacity of GST-HSA100. Warfarin is probably the most typical ligand for the subdomain IIa site, and the structures of the HSA complexes with both its enantiomers, having similar affinity, have been described [25]. Efavirenz, an inhibitor of HIV reverse transcriptase, is a widely used drug in AIDS therapy and has led to increased interest in the therapeutic drug monitoring of antiretroviral drugs. Efavirenz, as many drugs, binds to HSA and interacts with the subdomain II site [40]. The interactions of the two ligands with the construct were first studied by fluorescence quenching experiments monitoring tryptophan emission. The emission spectrum of apo-GST-HSA100 is shown in Figure **3b** (spectrum 1) as a synchronous scan at Δ = 60 nm and shows an overall maximum at 281 nm excitation (i.e., 341 nm emission), while the emission maximum of native HSA is at 340 nm [27]. Conversely, it is well known that the four GST tryptophan residues have poor emission in native GST with a maximum at 335 nm as they are buried in a hydrophobic environment [34].

Quenching of GST-HSA100 fluorescence (1 µM in phosphate buffer, pH 7.4) was observed in the presence of both warfarin and efavirenz. The effect of efavirenz on the synchronous emission spectrum is shown in Fig. **3b**, and similar results were obtained with warfarin (Figure S1). Quenching occurred and was accompanied by a blue shift of the excitation maximum from 281 to 278 nm. The highest quenching was obtained at 200 µM efavirenz, which is close to its solubility limit. In the difference spectrum between unquenched and quenched GST-HSA100 emission, there is a shoulder corresponding to an emission maximum at 330 nm. The blue shift from 341 nm upon titration with efavirenz is consistent with a change of the polarity of the environment surrounding the emitter Trp residue, resulting from replacement of the solvent in the active site by the less polar ligand. The buried, inaccessible, tryptophan residues in GST are likely to significantly contribute to the residual unquenched emission observed at 200 µM efavirenz (Fig. **3b**). The titration of fluorescence emission as a function of ligand concentration for HSA, GST-HSA100 and GST is reported in Figure **3c**. Both ligands had a similar quenching effect on HSA and GST-HSA100, whereas the effect on GST fluorescence was negligible (Fig. **3c**). Control experiments were carried out also with the ligand solvent (acetonitrile) alone, and no quenching was observed up to the final 1% concentration obtained at the highest added ligand amounts. To determine whether the observed quenching was due to binding or collisional phenomena, a Stern-Volmer analysis of the quenching data was performed in the 4300–24500 L mol^−1^ range (Table 1). Assuming a 5-ns decay time for tryptophan fluorescence, the apparent bimolecular quenching constants derived from the Stern-Volmer constants were as high as 8×10^11^–4.9×10^12^ l mol^−1^s^−1^ (Table 1) and 2–3 orders of magnitude higher than the limit for diffusion–limited collisional quenching. The latter can thus be excluded in favour of static quenching originating from the association of the fluorophore and quenchers in a bimolecular complex [23], [41].

**Table 1:** Binding parameters for warfarin and efavirenz.

	Warfarin		Efavirenz	
	HSA	HSA100	HSA	HSA100
**K_SV_** [a]	24.35±0.21	23.87±0.22	5.8±0.1	4.3±0.4
**k_q_** [b]	4.87±0.42	4.77±0.43	1.15±0.03	0.86±0.07
**K_D_** [c]	247±21	270±24	1.89±0.04	2.61±0.25
**n** [d]	1.07	1.02	0.98	0.98
**K_ass_** [e]			7.1±0.2	5.9±0.5

[a] Stern-Volmer quenching association constant (10^−3^Lmol^−1^).

[b] Bimolecular quenching kinetic constant (10^−12^Lmol^−1^s^−1^) assuming τ_0_ = 5 ns for the tryptophan fluorescence decay.

[c] Dissociation constant (10^−4 ^molL^−1^) for the protein-ligand complexes from Hill analysis of the quenching data.

[d] Number of binding sites per molecule of protein from Hill analysis of the quenching data.

[e] SPR steady state association equilibrium constant (10^−3^ Lmol^−1^).

The ligand–protein dissociation constants were evaluated by a Hill analysis of the fluorescence data (Table 1). The K_D_ values obtained for HSA and GST-HSA100 were similar and close to the literature values of 3.7–3.5 µM and 110 µM for warfarin [30], [42], [32] and efavirenz binding to native HSA [40], respectively. The binding of efavirenz to GST-HSA100 was also confirmed by a surface plasmon resonance experiment carried out on a Biacore instrument (Fig. S2). Efavirenz was modified at position 1 of the benzoxazin-2-one system to obtain derivative **2b** (Figure **3a**) and this amine was immobilized on a carboxymethylated dextran matrix attached to the gold chip. The affinity measured from the SPR data was comparable to that obtained by fluorimetry (Table 1).

Binding activity is therefore conserved in the GST-HSA100 fragment, and the measured affinities fit well with the hosting capacities of the native protein. Based on the fluorescence data, the affinity for free efavirenz in solution is similar to that measured for immobilized efavirenz by the SPR sensor. Efavirenz was immobilized to the sensor surface via its heterocyclic nitrogen, leaving the aromatic/cyclopropyl moieties of the molecule exposed for interaction with the protein. This is required by the shape of the IIa binding site, that contains two hydrophobic subsites specific for aromatic and hydrophobic groups.

To design a rapid screening methodology for binding by GST-HSA100, we performed the fluorescence-quenching titration in a 384 microwell plate format using a fluorimetric plate reader equipped with 280±15 nm excitation and 350±15 nm emission filters to detect tryptophan fluorescence. Due to the lower sensitivity of this method, microwell screening required a higher concentration of GST-HSA100 to provide a detectable fluorescence signal. The results are comparable with fluorimetric titrations in the spectrofluorimeter, enabling a considerably higher throughput and requiring less than a quarter of the volume (Figure S3). The development of this fast analysis method will also allow the rapid screening of libraries of mutated GST-HSA100 for improved binders.

### Chemical Reactivity Control: Diketone Reduction

**Figure 4:**
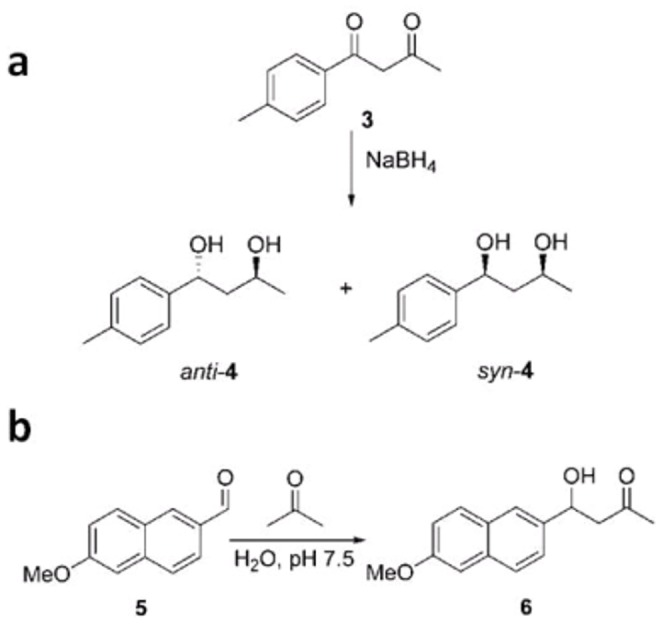
Control of reactivity with GST-HSA100. (**a**) Diastereoselective reduction of 1,3-diketones. (**b**) Albumin-catalyzed aldol addition.

1,3-Diols are naturally occurring compounds and important synthetic intermediates. Their preparation in diastereomerically pure form is a frequent target in organic synthesis, and has been achieved through several approaches, including biomimetic and organocatalytic methods. We found that *anti* 1,3-diol **4** can be obtained, with a diastereoisomeric excess of up to 96%, by the stereoselective reduction of diketone **3** (Figure **4a**) with sodium borohydride in aqueous acetonitrile in the presence of stoichiometric amounts of bovine or human albumin (Table 2) [20], [22]. The same reaction, without albumin, is not stereoselective, yielding a 1∶1 mixture of *syn* and *anti* diols **4**.

**Table 2:** Control of reactivity.

Protein	Diastereoselective reduction of diketone 3		Kinetic parameters (at 37°C) for the uncatalyzed, HSA and GST-HSA100-catalyzed aldoladdition of acetone to aldehyde 5				
	%anti	%syn	k_unc_ [a]	k_cat_ [b]	k_cat_/k_unc_	K_M_ [c]	k_cat_/K_M_k_unc_^[d]^
BSA	98	2					
HSA	88	12	8.04±0.53	1000±900	1244	2.1±0.2	5.9×10^5^
GST-HSA100	90	10	8.04±0.53	940±80	1170	3.0±0.3	3.9×10^5^

[a] observed pseudo first order value (10^6^ min^−1^).

[b] apparent value (10^6^ min^−1^) in 10% aqueous acetone.

[c] apparent value (10^3^molL^−1^). [d] Lmol^−1^.

When carrying out the reduction of diketone **3** in the presence of GST-HSA100, we found that the level of stereoselectivity was comparable to that obtained with native HSA (Table 2). Complete conversion of the substrate to *anti*-diol **4** takes place without loss of selectivity and the *anti* diol can be easily recovered from the aqueous medium by simple extraction after denaturing the peptide with ethanol.

The albumin directed diketone reduction takes place in two steps: [20] the more accessible aliphatic carbonyl, which is exposed to the solvent in the albumin-diketone complex, is reduced first in a completely chemoselective fashion. The aromatic carbonyl, buried inside the albumin binding site, is then reduced stereoselectively.

The ability of GST-HSA100 to efficiently control the same reaction is again consistent with a significant degree of structural conservation. Control requires the recognition of the 1,3-diketone, stabilization by ionizable residues H242 and K199 (native protein numbering) in their neutral and protonated forms, respectively, [20] and complete command over the two reduction steps. Clearly this can only be achieved if the overall shape of albumin’s binding site is conserved in the shorter peptide.

### Chemical Reactivity Control: Aldolase Activity

The aldol reaction is among the most important synthetic tools for the stereoselective formation of carbon-carbon bonds. Catalytic versions of this reaction have been proposed using metal catalysis, organocatalysis, and bio- and biomimetic catalysis. We have recently found that HSA and BSA catalyze the aldol addition of acetone to aldehyde **5** (Figure **4b**) and other aromatic aldehydes with an enzyme-like mechanism. The catalyzed process is three orders of magnitude faster than the uncatalyzed reaction, with Michaelis – Menten constants in the millimolar range [21].

**Figure 5:**
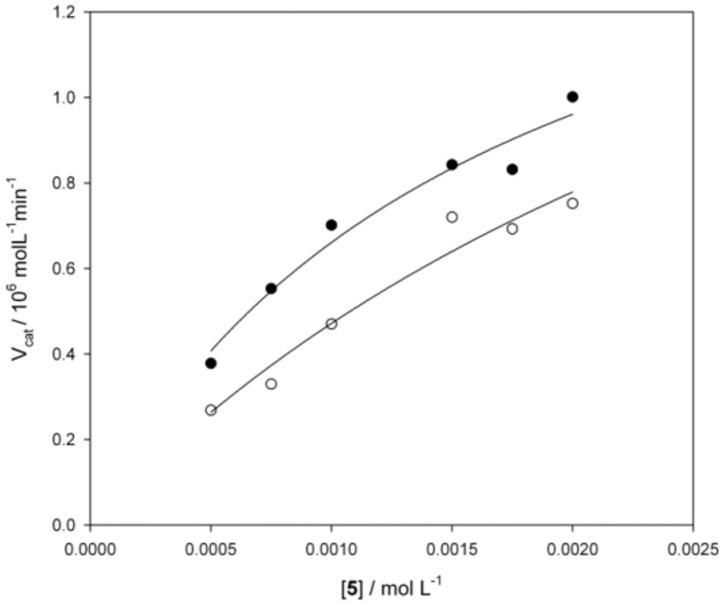
Aldolase activity of GST-HSA100. Michaelis–Menten plot of the initial velocities for the aldol addition of acetone to aldehyde **5** catalyzed by HSA (•) and GST-HSA100 (○).

GST-HSA100 behaved similarly to the parent protein (Fig. **5**, Table 2) accelerating the reaction by over 1100-fold. The construct exhibited multiple turnover, allowing more than 100 catalytic cycles and full conversion of a 100-fold excess of aldehyde **5** to aldol **6**.

Experimental evidence supports the hypothesis that the HSA-catalyzed aldol reaction occurs through an enamine intermediate via a mechanism similar to that of type 1 aldolases. Lysine 199 reacts with acetone to produce the acetone enamine, which acts as nucleophile toward the aldehyde substrate. The conservation of aldolase activity in the peptide suggests that the peculiar pKa of this key lysine residue is not significantly altered in the shortened protein sequence. These data unveil the possibility of successfully obtaining aldolase peptides with enhanced efficiency and stereoselectivity from mutated libraries of GST-HSA100.

### Conclusion

Despite the fact that the CD and MS data presented in this paper are just consistent with, but not ultimately proving a correct folding of the albumin fragment, the protein has proven to retain the binding and catalytic activities of the whole albumin. We are currently working in order to obtain the peptide alone after cleavage of GST, and to study its structure. Nevertheless, we believe that GST-HSA100 is a promising scaffold for the engineering of binders and catalysts with optimized properties and may provide an alternative to conventional antibodies as library templates for phage display. To this end, the expression of GST-HSA100 in phages is currently being studied in our laboratories.

## Methods

### Materials

HSA essentially free of fatty acids (A3782), (±)-warfarin (A2250) and all other reagents were purchased from Sigma–Aldrich. SPR reagents, buffers and the research-grade Sensor Chip CM5 were obtained from GE Healthcare Bio-Sciences. Efavirenz was synthesized as previously reported [43]. Phosphate buffer (10 mM Na_2_PO_4_, 1.76 mM KH_2_PO_4_, pH 7.4) was used to perform all CD and fluorescence experiments. PBS was used in SPR analyses.

### Synthesis of the Efavirenz Derivative 2b

Efavirenz was first reacted with chloroacetic acid to obtain its N-carboxymethyl derivative [44]. This compound was then coupled with N-Boc-1,5-diaminopentane [45] to give ***tert*-butyl-5-(2-((*R*)-6-chloro-4-(2-cyclopropylethynyl)-4-(trifluoromethyl)-2-oxo-2*H*-benzo[*d*] [1], [3]oxazin-1(4*H*)-yl)acetamido)pentylcarbamate:** the acid analogue of efavirenz (190 mg, 0.51 mmol)) was stirred in dry CH_3_CN (3.4 mL) under argon atmosphere conditions. HOBt (69 mg, 0.51 mmol) and NMM (about 112 µL, 1.02 mmol) were added maintaining pH at 8–8.5. The mixture was cooled to 0°C, EDC-Cl (117 mg, 0.61 mmol) and *N*-Boc-cadaverine (103 mg, 0.51 mmol) were added. The mixture was stirred for 1 h at 0°C then was allowed to reach room temperature and stirred for additional 16 hours. Then the solvent was removed by distillation under reduced pressure and the crude mixture was partitioned between ethyl acetate and water, the organic layer was washed with aqueous solution of citric acid 10% (w/v), saturated solution of NaHCO_3_, saturated solution of NaCl and dried over anhydrous Na_2_SO_4_. The solvent was removed to regain the compound as a white solid (171 mg, 60%). ^1^H NMR (400 MHz, CDCl_3_) δ 7.54 (*d*, 1H, *J = *1.8 Hz CClCHCC(CF_3_)), 7.43 (*dd*, 1H, *J_1_ = *8.8 Hz, *J_2_ = *2.0 Hz, CClCHCHN), 7.10 (*d*, 1H, *J = *8.7 Hz, CClCHCHCN), 6.30 (*br*, !H, CH_2_(C = O)NH), 4.44 (*AB*, 2H, *J_AB_ = *16.2 Hz, NCH_2_CO), 3.22 (*m*, 2H, ((C = O)NHCH_2_), 3.06 (*m*, 2H, (CH_2_NH(C = O)OC(CH_3_)_3_), 1.42 (*m*, 16H, (CH_3_)_3_, (C = O)NH(CH_2_)_3_, CH(CH_2_)_2_), 0.96–0.85 (*m*, 4H, CH(CH_2_)_2_); ^13^C NMR (100 MHz, CDCl_3_) δ 166.89 (COO(CH_3_)_3_), 156.46 (CH_2_(C = O)NHCH_2_), 149.07 (O(C = O)N), 135.15 (CHCN), 132.05 (CClCHCH), 129.93 (CCl), 128.18 (CClCHC), 122.80 (CCCF_3_), 120.84 (CF_3_), 117.51 (CHCHCN), 116.07 (CCF_3_), 95.36 (C≡CCH), 65.88 (C≡CCH), 49.23 (CH_2_(C = O)NH), 40.37 (CH_2_NH(C = O)OC(CH_3_)_3_), 39.62 ((C = O)NHCH_2_), 29.71–29.01 (C = O)NH(CH_2_)_3_, 28.54 (O(CH_3_)_3_), 8.99 (CH(CH_2_)_2_), −0.49 (CH(CH_2_)_2_; IR (cm^−1^) 3583, 3583, 3362, 2935, 2831, 2255, 1723, 1495, 1201, 1029; MS (ESI, m/z) 558 [MH]^+^. The compound was N-deprotected by stirring in 50% TFA/CH_2_Cl_2_ for 30 min at rt. The solvents were removed under vacuum. The solid residue obtained was dried under vacuum to obtain brown solid **2b** (125 mg, 100% yield), ***N*** **-(5-aminopentyl)-2-((** ***R*** **)-6-chloro-4-(2-cyclopropylethynyl)-4-(trifluoromethyl)-2-oxo-2** ***H*** **-benzo[** ***d*** **]** [**1]**, [3] **oxazin-1(4** ***H*** **)-yl)acetamide** [α]_D_ = −22 (c = 0.3, CH_3_OH)**.** ^1^H NMR (500 MHz, CDCl_3_) δ 7.65 (*br*, 3H, CH_2_NH_3_ ^+^), 7.50 (*d*, 1H, *J = *1.7 Hz CClCHCC(CF_3_)), 7.40 (*dd*, 1H, *J_1_ = *8.8 Hz, *J_2_ = *2.0 Hz, CClCHCHN), 6.89 (*d*, 1H, *J = *8.7 Hz, CClCHCHCN), 6.38 (*br*, 1H, CH_2_(C = O)NH), 4.50 (*AB*, 2H, *J_AB_ = *16.6 Hz, NCH_2_CO), 3.20 (*m*, 2H, ((C = O)NHCH_2_), 2.93 (*m*, 2H, (CH_2_NH_3_^+^), 1.63 (*m*, 2H, CH_2_CH_2_NH_3_^+^), 1.47 ((C = O)NHCH_2_CH_2_), 1.34 (*m*, 4H, CH_2_CH_2_CH_2_NH_3_^+^, CH(CH_2_)_2_), 0.93–0.78 (*m*, 4H, CH(CH_2_)_2_); ^13^C NMR (125.4 MHz, CDCl_3_) δ 167.31 (COO(CH_3_)_3_), 161.43 (CH_2_(C = O)NHCH_2_), 149.20 (O(C = O)N), 135.23 (CHCN), 131.97 (CClCHCH), 129.71 (CCl), 128.05 (CClCHC), 123.39 (CCCF_3_), 121.11 (CF_3_), 117.09 (CHCHCN), 115.70 (CCF_3_), 95.40 (C≡CCH), 65.89 (C≡CCH), 47.46 (CH_2_(C = O)NH), 39.88 (CH_2_NH_2_), 39.29 ((C = O)NHCH_2_), 28.23 ((C = O)NHCH_2_ CH_2_), 26.68 (CH_2_CH_2_NH_2_), 23.29 (CH_2_CH_2_CH_2_NH_2_), 8.89–8.87 (CH(CH_2_)_2_), −0.64 (CH(CH_2_)_2_; IR (cm^−1^) 3583, 3018, 2928, 2250, 1729, 1498, 1198; MS (ESI, m/z) 458 [MH]^+^, 480 [MNa]^+.^

### Cloning of the HSA Binding Site II

The HSA binding site II domain sequence was designed with a codon optimization strategy for *E. coli* expression and ordered from Invitrogen GeneArt. The optimized sequence was subcloned from the original pMA vector into the prokaryotic expression vector pGEX 4T-1 (GE Healthcare) and ligation was transformed on Rosetta-gami B strain (Novagen) to yield GST fusion products. All cloning was verified by sequencing.

### GST-HSA100 Production and Purification

Colonies harbouring the plasmid were grown, and GST-HSA100 was expressed and purified as previously reported [46] with slight modification. Briefly, cells were grown to an OD of 0.6 at 600 nm and induced overnight with 0.2 mM IPTG at 25°C. Bacteria were harvested by centrifugation, and the cell pellets were lysed with 10 ml of lysis solution for each gram of bacteria, containing lysis buffer (20 mM Tris pH 8.00, 500 mM NaCl, 0.1% Triton X-100), 400 µg/mL lysozyme, 50 µg/mL DNAse, and a protease inhibitor cocktail. After centrifugation, the supernatants were collected and purified by affinity chromatography using Glutathione Sepharose 4B (GE Healthcare) according to the manufacturer’s instructions. Fractions were subsequently pooled and dialyzed in phosphate buffer or PBS overnight at 4°C. Protein production and purity was verified by SDS-PAGE followed by Coomassie Blue staining and Western blotting using anti-FLAG antibody.

### CD Spectroscopy

CD spectra were obtained on a Jasco J-710 spectropolarimeter. Spectra were recorded in a 0.2-cm quartz cuvette at temperatures ranging from 5 to 75°C. Data were collected with a data pitch of 0.2 nm, a scanning speed of 50 nm/min and a bandwidth of 1 nm. Each spectrum was the average of 10 scans. A 1.35 µM stock solution of GST-HSA100 was prepared and diluted to 135 nM for CD measurements.

### Fluorescence

Warfarin and efavirenz stock solutions were prepared in acetonitrile. Steady-state fluorescence spectra were acquired at 298 K on a CARY Eclipse (Varian) spectrofluorimeter. Synchronous fluorescence spectra were measured in the 240–320 nm excitation range, and the emission was recorded at Δ = 60 nm. The concentration of protein (HSA, GST, or GST-HSA100) was maintained at 1 µM in 350 µl phosphate buffer, whereas the concentration of warfarin or efavirenz was gradually increased (from 1 to 300 µM and 1 to 2 mM for warfarin and efavirenz, respectively). After the addition of each ligand, the fluorescence intensity at the maximum emission wavelength and the drift of such maxima were measured after equilibrium had been reached (15 min).

### SPR Analysis

The efavirenz derivative **2b** was dissolved at a 1 mM concentration in 1∶10 borate buffer (10 mM disodium tetraborate, 1 M NaCl) with 5% of DMSO. Derivative **2b** was then coupled to Sensor chip CM5 using an EDC/NHS coupling kit in 1∶10 HBS-EP buffer [0.1 M HEPES, 1.5 M NaCl, 30 mM EDTA, 0.5% v/v p20 surfactant]. Excess active esters were deactivated with ethanolamine. The coupling reactions were performed using a flow rate of 10 µl/min for 7 min, resulting in densities that were approximately 400±50 RU on average. Analyses of interactions were carried out with a constant flow rate of 10 µL/min in PBS (53 mM Na_2_HPO_4_.7H_2_0, 12.5 mM KH_2_PO_4_, 70 mM NaCl, pH 7.4) as running buffer with an optimal GST-HSA100 concentration of 5 µM. After each cycle, both surfaces were regenerated with 30% acetonitrile in 1 mM NaOH. The reference data from a deactivated flow cell were subtracted from the sensorgrams, and the interaction equilibrium constant was calculated using the BIACORE X100 evaluation software.

### HPLC

Analyses of the reduction of diketone **3** and the aldol addition of acetone to aldehyde **5** were performed on a HP1100 series instrument equipped with a C18 Phenomenex Luna column (5 µm, 150×4.60 mm) using appropriate mixtures of acetonitrile and water as eluent. A flow rate of 0.5 ml/min was used, and the reaction products were monitored at 214 nm. Diketone **3** and its *anti* and *syn* diols **4** were separated using a 68∶32 water/acetonitrile mixture. Aldehyde **5** and its aldol product **6** were separated using a 40∶60 water/acetonitrile mixture.

### Reduction of Diketone 3

A solution (0.5 ml) of diketone **3** (43 µM) in acetonitrile was added to 0.5 ml of 43 µM GST-HSA100 in water, and the resulting mixture was maintained at room temperature for 30 min. A solution (0.5 ml) of NaBH_4_ (75 µM) in water was then added at 20°C, and the resulting mixture was stirred at the same temperature for 2 h. The mixture was acidified with trifluoroacetic acid (150 µl), and 1 ml ethanol was added. Next, the mixture was filtered with a 0.22-µm filter and analyzed by HPLC.

### Aldol Reaction

GST-HSA100 (200 µM) in 90 µL of phosphate buffer (0.05 M Na_2_HPO_4_, 0.5 M NaCl, pH 7.5) was incubated at 37°C for 15 min. A solution of aldehyde **5** (2 µl) in acetonitrile was added for final concentrations ranging from 0.5 to 2 mM followed by the addition of 10 µl of acetone. The samples were subsequently sealed and kept at 37°C. Initial velocities of the aldol reaction were obtained by measuring the concentrations of aldehyde and aldol products by HPLC, while the reactions went to 5% completion.

## Supporting Information

Figure S1 **Synchronous (Δ = 60 nm) fluorescence spectra of GST-HSA100 1 µM in 10 mM phosphate buffer, pH 7.4. 1, no quencher added; 2, 60 µM warfarin; 3, 100 µM warfarin; 4,200 µM warfarin.**
(TIF)

Figure S2 **SPR sensogram for binding of GST-HSA100 to an efavirenz-coated surface.**
(TIF)

Figure S3 **Fluorescence-quenching titration in a 384-microwell plate format using a fluorimetric plate reader equipped with 280±15 nm excitation and 350±15 nm emission filters to detect tryptophan fluorescence.** GST-HSA100 was 20µM.
(TIF)

Table S1 (DOCX)
