# Supplementary figures and images for: An Albumin-Derived Peptide Scaffold Capable of Binding and Catalysis
Source: PLoS One. 2013 Feb 22;8(2):e56469. doi: 10.1371/journal.pone.0056469 (PMC3579865; doi:10.1371/journal.pone.0056469)

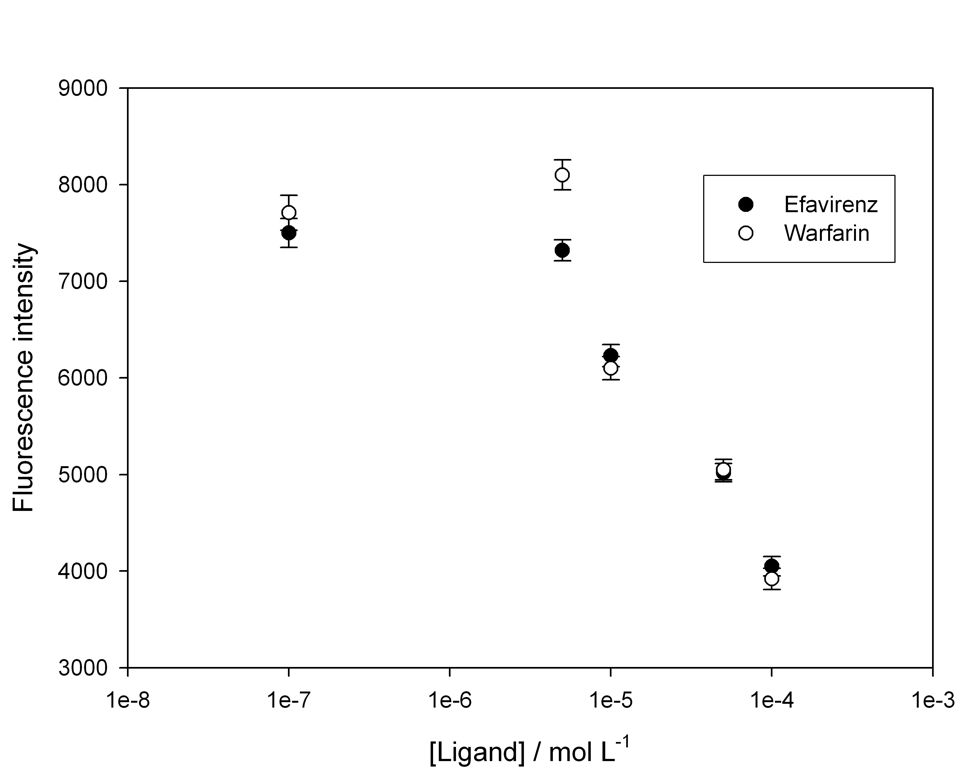

Supplement: Figure S3 — Fluorescence-quenching titration in a 384-microwell plate format using a fluorimetric plate reader equipped with 280±15 nm excitation and 350±15 nm emission filters to detect tryptophan fluorescence. GST-HSA100 was 20µM. (TIF) [file pone.0056469.s003.tif]
