# Supplementary material for: An Albumin-Derived Peptide Scaffold Capable of Binding and Catalysis
Source: PLoS One. 2013 Feb 22;8(2):e56469. doi: 10.1371/journal.pone.0056469 (PMC3579865; doi:10.1371/journal.pone.0056469)
Supplement: Table S1 — (DOCX) [file pone.0056469.s004.docx]

| **Supplementary Table 1: GST-HSA100** peptides detected by mass spectrometry | | | | | | | | |
| --- | --- | --- | --- | --- | --- | --- | --- | --- |
| **Peptide** | **Molecular Mass** | | **Elution (min)** | **m/z^1+^** | **m/z^2+^** | **m/z^3+^** | | **Sequenced (MS/MS)** |
|  | th. | exp. |  |  |  |  |  |  |
| 196-197 | 302.17 | 302.2 | 3.8 | 303.2 |  |  | |  |
| 200-205 | 648.32 | 648.5 | 11.4 | 649.3 | 325.2 |  | |  |
| 206-209 | 507.24 | 507.3 | 8.5 | 508.3 |  |  |  | |
| 200-209 | 1137.56 | 1137.6 | 19.7 | 1138.5 | 569.8 | 380.2 | | X |
| 210-212 | 364.21 | 364.2 | 5.1 | 365.2 |  |  | |  |
| 213-218 | 672.37 | 672.4 | 19.9 | 673.4 | 337.2 |  | | X |
| 219-222 | 502.28 | 502.3 | 4.5 | 503.3 |  |  | |  |
| 223-225 | 390.22 | 390.2 | 10.6 | 391.2 |  |  | |  |
| 226-233 | 879.43 | 879.4 | 19.4 | 880.4 | 440.7 |  | | X |
| 234-240 | 788.46 | 788.5 | 20.2 | 789.5 | 395.3 |  | | X |
| 258-262 | 516.29 | 516.3 | 5.9 | 517.3 |  |  | |  |
| 263-274 | 1385.61 | 1385.6 | 20.2 |  | 693.8 |  | | X |

**Supplementary table 1** GST-HSA100 LC-MS/MS identification.
